# Ovarian borderline tumor presenting as ovarian torsion in a 17-year-old patient: a case report

**DOI:** 10.1186/s13256-020-02597-1

**Published:** 2021-01-12

**Authors:** Ana Patrícia Vicente, Andrea Sousa Gomes, Ligita Jokubkiene, Povilas Sladkevicius

**Affiliations:** 1 Department of Obstetrics and Gynecology, Hospital de Cascais - Dr. José de Almeida, Lisbon, Portugal; 2 Mälmo Department of Obstetrics and Gynecology, Skåne University Hospital, Lund University, Mälmo, Sweden

**Keywords:** Ovarian torsion, Ovarian borderline tumors, Adolescent, Ultrasonography, Case report

## Abstract

**Background:**

Ovarian torsion is a gynecological surgical emergency whose diagnosis remains a challenge. Torsion occurs most frequently in women of reproductive age. It is usually associated with the presence of benign masses in the ovary, as malignant tumors are less frequent and less prone to undergo torsion.

**Case presentation:**

We report the case of a 17-year-old Caucasian patient who presented to the emergency department with lower abdominal pain. Ultrasonography evaluation revealed a unilateral ovarian lesion, 11.2 cm, with features suspicious for malignancy and torsion. The patient was referred for surgical torsion treatment and underwent unilateral salpingo-oophorectomy. The pathology report confirmed a serous borderline ovarian tumor with torsion.

**Conclusions:**

Malignant ovarian torsion in pediatric age groups is rare. Ultrasound examination should be recognized as a powerful tool for diagnosis and management, especially when performed by an experienced ultrasonographer.

## Background

Ovarian torsion results from partial or complete rotation of the ovary around its axis, leading to obstruction of the vascular pedicle [1, 2]. Although it occurs in normal ovaries, most frequently it is related to increased ovarian volume and benign masses [1–3].

Most ovarian masses in pediatric and adolescent patients are benign. A study of surgically managed ovarian masses in patients up to 19 years of age revealed that 2.3% were borderline and 5.3% were malignant, with no reported cases of ovarian torsion in either of these groups [4].

The rate of malignancy in ovarian torsion is low. When compared with malignant ovarian neoplasms, benign lesions have a 12.9-fold increased risk of being involved in adnexal torsion [5]. Diagnosis of ovarian torsion is challenging, since clinical features can be nonspecific and variable. The majority of patients present with lower quadrant pain, nausea and vomiting, which can mimic many other causes of abdominal pain [6, 7]. A palpable abdominal mass can also be present, a feature that is significantly associated with malignant masses [4].

Transvaginal sonography (TVS) is widely used for the evaluation of adnexal masses, and remains the primary diagnostic modality for suspected ovarian torsion [8, 9]. It is an accurate method for discriminating between benign and malignant lesions, especially when performed by an experienced ultrasonographer [10]. Advances in technology and the use of color Doppler have been helpful in the diagnosis of ovarian torsion, since they have increased diagnostic accuracy and positive predictive value [1, 11].

Less frequent and unexpected findings sometimes appear together. Proper, accurate and complete preoperative evaluation is always essential, ensuring the best possible outcome for each patient.

We report a case of ovarian torsion with a serous borderline ovarian tumor in a pubertal-aged girl.

## Case presentation

A 17-year-old Caucasian girl presented to the emergency department complaining of acute lower abdominal pain. The pain started suddenly, 2 hours before, in the mid-right lower abdomen. She also complained of diarrhea earlier that day, but no other symptoms. The patient had no remarkable medical history, no surgeries, no medication other than hormonal contraceptive pills, and no previous pregnancies. The pain remained constant, without relief from nonsteroidal anti-inflammatory treatment.

On admission she was hemodynamically stable, no fever, with lower mid/right abdominal pain on palpation, without peritoneal reaction.

**Fig. 1:**
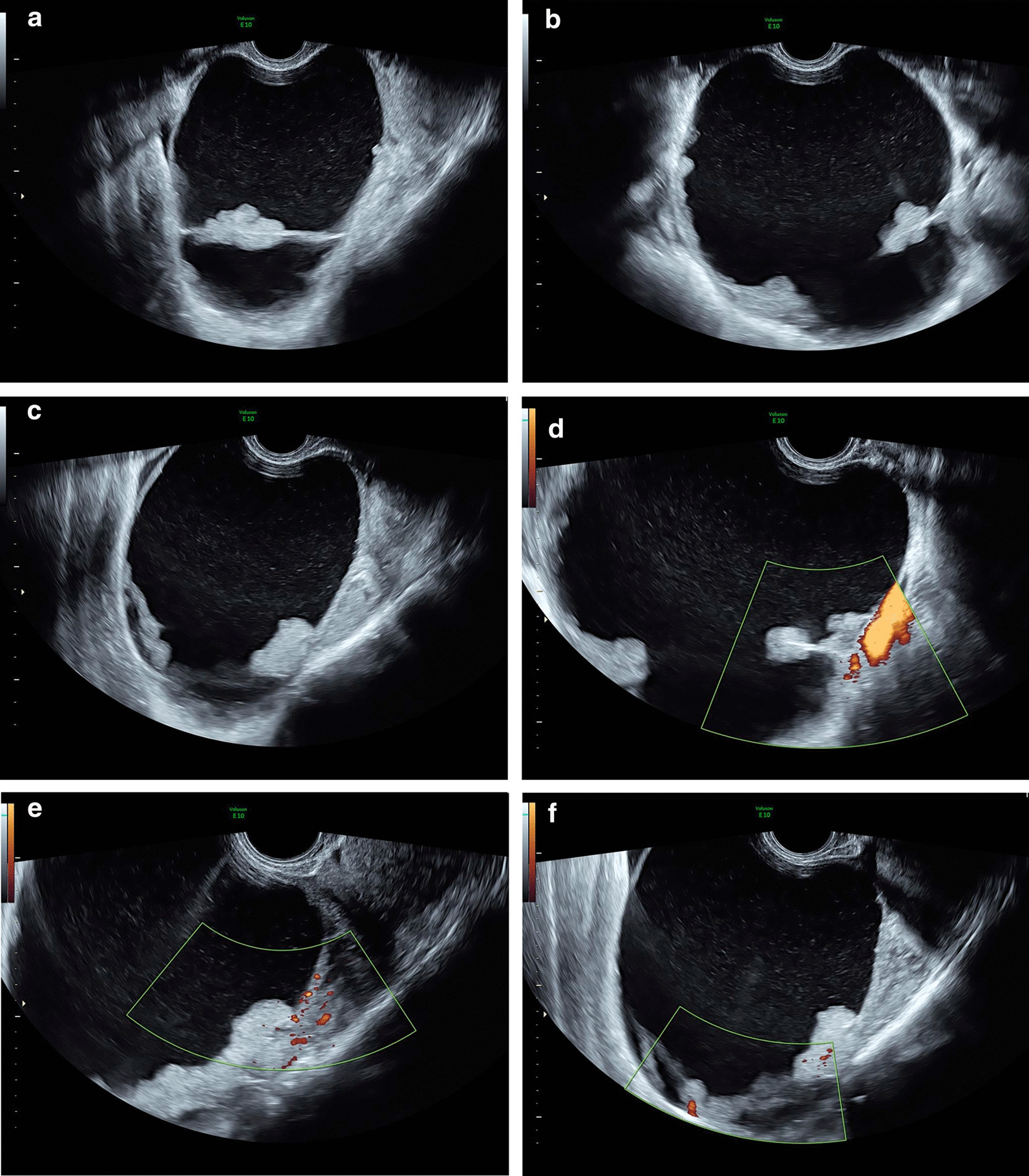
Grayscale (**a**–**c**) and color Doppler (**d**–**f**) transvaginal sonography: right ovary multilocular-solid mass, with papillary excrescences and low vascularization

**Fig. 2:**
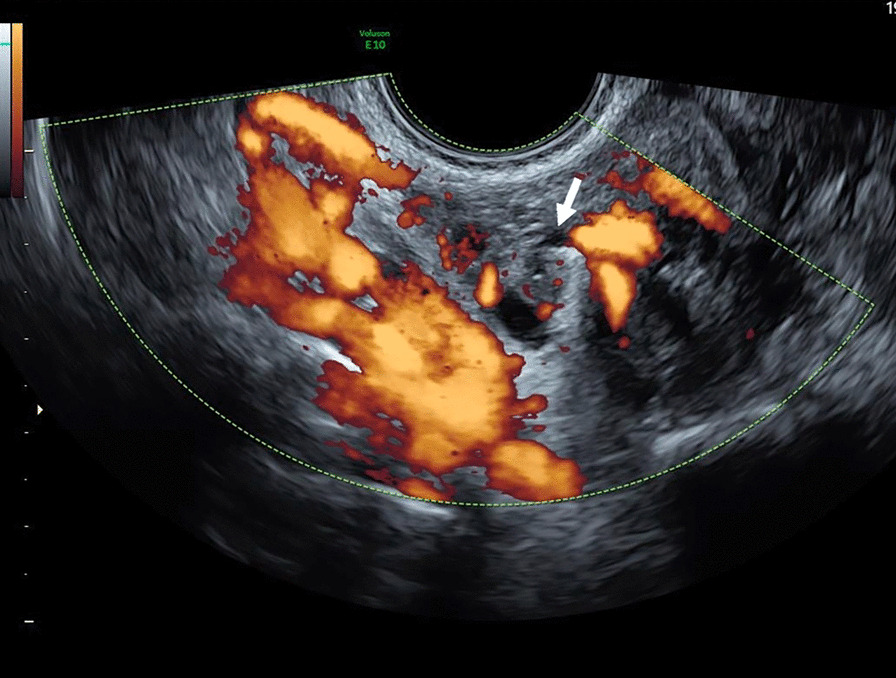
Color Doppler transvaginal ultrasonography: twisted ovarian pedicle (arrow)

**Fig. 3:**
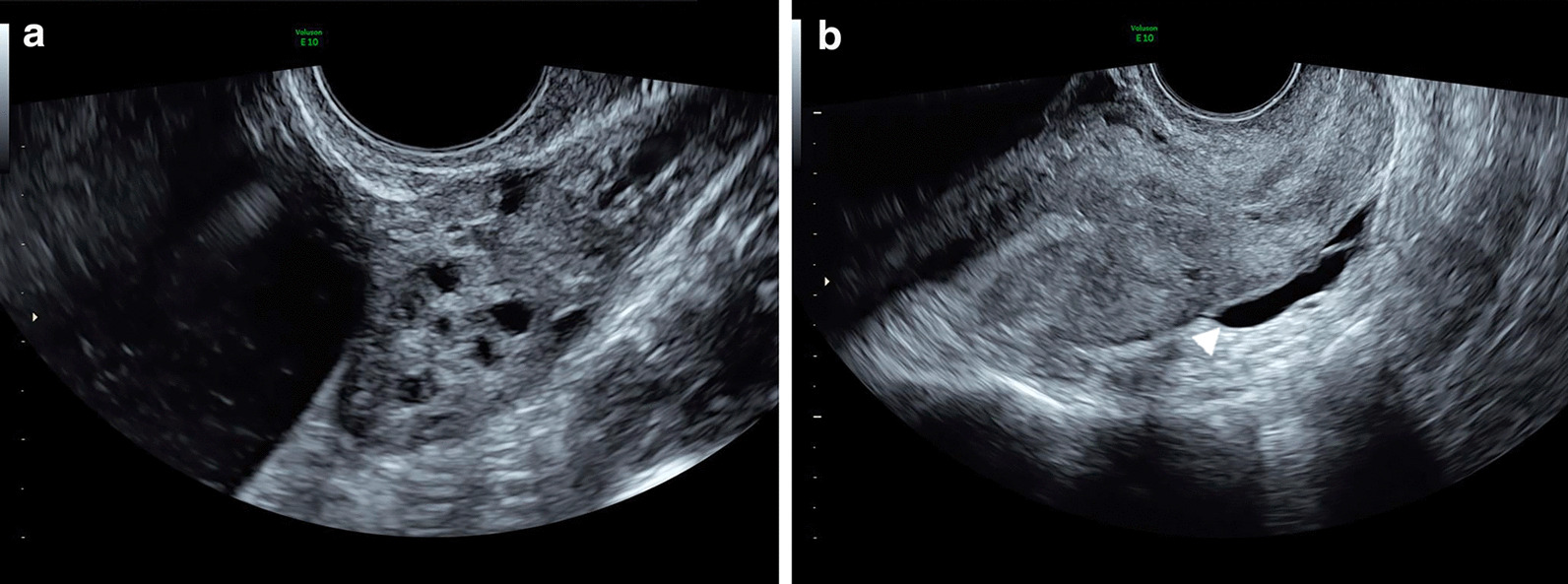
Grayscale transvaginal ultrasonography: left ovary (**a**) and uterus (**b**). Fluid in the pouch of Douglas (arrowhead)

Gynecological evaluation revealed a soft mass 10 cm in size in the right lower abdominal quadrant, and no other signs. Laboratory evaluation showed no anemia, no inflammatory parameters and negative urinary pregnancy test (hemoglobin 12.2 g/dL; C-reactive protein [CRP] 3.7 mg/L). She was then referred to the ultrasound department for transvaginal ultrasound evaluation, which revealed a right multilocular solid mass, measuring 10.8 × 10.2 × 12.5 cm, with increased vascularization and tenderness, suspicious for borderline tumor with torsion (Figs. 1, 2 and 3).

She was referred for emergency laparoscopy, and a right salpingo-oophorectomy was performed. Recovery, hospital discharge and postoperative follow-up were uneventful. The final histopathology analysis confirmed torsion of a serous borderline ovarian tumor.

## Discussion and conclusions

Torsion is reported in 1.1–2% of malignant ovarian neoplasms [5, 12]. A 15-year analysis of 707 patients with proven ovarian torsion reported 13 malignancies: eight juvenile granulosa cell tumors, four dysgerminomas, four borderline tumors (three serous and one mucinous) and two undifferentiated adenocarcinomas. To our knowledge there are only a few reports of torsion in borderline serous tumors, and only two in patients under the age of 19 [3].

Borderline tumors, commonly described as having low malignant potential, present papillary projections and increased cell proliferation rate, but absent stromal invasion [13, 14]. Borderline tumors are often diagnosed in the early stage [14]. They are most typically are seen in patients 10 years younger than those with other ovarian malignancies, and one third are in women less than 40 years of age [15]. The prognosis is usually good. For stage I disease, the 5-year survival rate is 95–97%, while 10-year survival is 70–95%, because of late recurrence [16]. The most common histological type is serous (50%), which presents as bilateral in 30% of cases [14].

Borderline ovarian tumors are asymptomatic in 30% of cases, with nonspecific symptoms in 50–60% of cases [17]. Most borderline tumors are detected incidentally by ultrasound [16], which is recognized as an accurate method for distinguishing between benign and malignant adnexal masses [10].

It is reported that malignant lesions have a low probability of undergoing torsion because of their propensity to adhere to local structures, due to inflammation, adhesions or local invasion [5]. Whether this applies to borderline tumors is not yet clear, although the scarcity of such presentation could point to some similarity in pathological behavior.

Ovarian torsion is one of the most common gynecological surgical emergencies [12]. It is more frequently seen in women of reproductive age, with nearly 75% developing in women between the ages of 20 and 40 (mean 32–33.5) years, although it can occur at any age [6, 7]. Our patient was 17 years old. With regard to the pediatric population, one of the largest reviews conducted found that 52% of torsions occurred in patients between the ages of 9 and 14 years, as a result of increased hormone levels during this period [18].

Early diagnosis is extremely important in any circumstance, but is most significance in young women, since it may dictate fertility preservation [19, 20]. The preferred use of conservative treatment (detorsion and avoidance of salpingo-oophorectomy) has slowly increased over the past 20 years, to 45%, as it is considered a safe option [21, 22]. In the past, some concerns have arisen regarding this approach, often driving the choice of proceeding with oophorectomy. The risk for pulmonary embolism, difficult evaluation of ovary viability and the possibility of an underlying malignancy in an enlarged and edematous ovary are some of the main issues [18].

Malignant tumors are a rare underlying cause of torsion [12, 23], and most pathological ovarian masses in the pediatric population are benign. However, failure to suspect or detect a malignant tumor may compromise patient prognosis [4]. One of the largest reviews performed in the pediatric population found a greater likelihood of malignancy in torsion if the mass at presentation was greater than 8 cm [3].

In light of the above, despite the increasing trend toward ovarian preservation in ovarian torsion, this may not be the proper approach for all, even at a young age. Adequate assessment of adnexal masses in cases of torsion, even during urgent patient examination, cannot be disregarded. Different and more aggressive management may be needed if malignancy is considered.

TVS evaluation is important and the first-choice imaging technique in many centers [24]. It is well known that the diagnosis of ovarian torsion is extremely challenging. Not infrequently, patients can have subtle or nonspecific clinical symptoms, making differential diagnosis difficult [6, 7].

Torsion can be partial or complete, with several degrees of compromised circulation. As a result, the sonographic appearance is affected by the duration and degree of torsion [1, 25, 26].

The most common sonographic finding is the presence of an enlarged ovary, with or without a mass, and fluid in the cul-de-sac, which is usually a late manifestation [27]. Color Doppler for diagnosis of torsion remains controversial because of the dual blood supply to the ovary [22]. Sometimes an ovarian twisted pedicle can be identified (“whirlpool sign”) [1].

The value of ultrasonography cannot be overemphasized, especially when performed by an experienced examiner. However, it is imperative to bear in mind that even a more common diagnosis, such as ovarian torsion in a younger patient, can uncover surprising histopathological findings such as borderline and malignant ovarian tumors. In the present case, ultrasound examination was first requested to assess the likelihood of torsion as the cause of the clinical presentation. Proper evaluation revealed signs of both suspected torsion and suspected malignancy. In addition to the suggestion of laparoscopy for torsion, the high suspicion regarding the malignant character of the lesion prompted a less conservative intervention, even at a young age, diminishing the need for a second surgery.

Ultrasonography adds increased accuracy and positive predictive value for ovarian torsion diagnosis, and the ability to discern benign from malignant lesions, especially when performed by an experienced ultrasonographer. At all times, ultrasound examination should be recognized as a powerful tool within a comprehensive diagnostic and management approach.

## Data Availability

All data generated or analyzed during this study are included in this published article.

## Abbreviations

TVS: Transvaginal sonography
CRP: C-reactive protein
